# Oral and nasal provocation test in chicken egg allergy—case report

**DOI:** 10.1186/s13223-023-00829-9

**Published:** 2023-08-14

**Authors:** E. Krzych-Fałta, O. Wojas, P. Samel-Kowalik, B. Samoliński, A. Sybilski, S. Białek

**Affiliations:** 1 Department of Basic of Nursing, Medical University of Warsaw, Warsaw, Poland; 2 Department of Prevention of Environmental Hazards, Allergology and Immunology, Medical University of Warsaw, Banacha 1a Str, 02097 Warsaw, Poland; 3 Second Department of Pediatrics, Center of Postgraduate Medical Education, Warsaw, Poland; 4 Department of Biochemistry and Pharmacogenomics, Medical University of Warsaw, Warsaw, Poland

**Keywords:** Food allergy, Egg allergy, Placebo-controlled food challenge, Nasal provocation test, Nasal mucosa reactivity

## Abstract

**Introduction:**

Allergy to chicken egg protein is a common form of food allergy. The most common clinical presentation includes gastrointestinal, skin, and respiratory symptoms. Differential diagnosis, including provocative tests, is critical in diagnosis.

**Case description:**

We present a case of a 21-year-old patient with egg allergy, who underwent a double-blind food provocation test with placebo (evaluating subjective complaints from the gastrointestinal tract) and a titrated nasal provocation test using dry chicken egg content. We assessed the response of the nasal mucosa in the provocation test using the visual analogue scale (VAS), acoustic and optical rhinometry, as well as measurements of nitric oxide concentration in the exhaled air. During the provocation test, we measured the changes in the transverse section of the nasal passages, which were accompanied by subjective complaints measured with the VAS scale, using objective techniques. In the nasal provocation test with a dose of 20 µg of chicken egg protein, we observed an increase in the reactivity of the nasal mucosa and a decrease in the level of nitric oxide in the exhaled air from the upper airways (920 ppb before the provocation test and up to 867 ppb during the early stage of the allergic reaction). During the provocation tests, we recorded typical symptoms associated with the early stage of the allergic reaction; including nasal obstruction (1.2 cm), leakage of watery discharge (0.8 cm) in the food test, and itchy nose (1.1 cm) in the food test vs. the nasal test: 4.6, 2.8, and 3.5 cm, respectively.

**Conclusions:**

The nasal mucosa provides convenient conditions for evaluation of the severity of allergy to common food allergens, including chicken egg allergens.

## Introduction

Food allergy is a constellation of symptoms occurring every time after exposure to a given food at a dose tolerated by healthy individuals. Food allergy, unlike food intolerance, is an IgE-dependent or IgE-independent adverse reaction to food mediated by the immune system [1, 2]. Pathophysiology of food allergy is based on complex interactions between the gastrointestinal mucosa, local and systemic immune reactivity, and microbiome [1, 2]. The incidence of food allergy is increasing worldwide and is becoming a serious public health issue. Currently, the incidence of food allergy is estimated at 6 to 10% in the pediatric population and 2 to 5% in adults. More than 220 million people worldwide suffer from food allergy. Although there is a lack of precise epidemiological studies, the incidence of food allergy seems to have increased substantially over the past two decades in Western countries and is ca. 10% in preschool children [2–4]. Diagnosis of food allergy includes detailed history, physical examination, skin tests (prick, native, patch tests), laboratory tests [specific IgE, component resolved diagnosis (CRD), basophil activation test (BAT)], and elimination diets. However, the most important study and the gold standard in the diagnosis of food allergy remains a double-blind, placebo-controlled food challenge (DBPCFC) [1, 5]. The primary indication for the provocation test is the need to confirm the cause-and-effect relationship between the consumption of a given food and the hypersensitivity reaction. The provocation test reproduces and mimics the body’s natural response to the given allergen. A positive result of the provocation test is usually decisive for introducing an elimination diet. It is important to note that any diagnostic provocation test carries a potential risk of triggering bothersome or even dangerous symptoms to the patient. Considering the risk of anaphylaxis, such tests should be performed by an allergologist accompanied by an appropriately trained medical personnel in a hospital setting as per e.g. Polish guidelines [5, 6]. The clinical presentation of food allergy is extremely diverse and depends on the type of food, patient’s age, and personal predisposition. Undoubtedly, the most common manifestations are symptoms from the gastrointestinal tract; each part of the digestive tract can be affected. During oral provocation tests with an allergen, patients present nasal symptoms such as itching, sneezing, watery discharge, and nasal obstruction [1, 7]. Those observations have laid the foundation for further research on the use of nasal provocation tests in the diagnosis of food allergy. The nasal provocation test (NPT) is widely used in the diagnosis of rhinitis. It allows us to confirm the causal role and identification of factors triggering an IgE-dependent response in allergic rhinitis, and to confirm the effectiveness of pharmacotherapy and specific immunotherapy in the treatment of allergic rhinitis. NPT is a relatively safe procedure and can be performed in an outpatient clinic. Symptoms of an immediate reaction are usually no longer evident after a dozen or so minutes. Interpretation of the provocation test requires clinical evaluation and objective techniques such as rhinomanometry, acoustic and optical rhinometry (AR, OR), as well as peak nasal inspiratory flow (PNIF) [6–8]. An alternative to those nasal patency tests during NPT is e.g. the level of nitric oxide in exhaled air as an inflammatory reaction marker [9]. The main objective of this study was to conduct an experiment evaluating nasal mucosal involvement during NPT and DBPCFC with chicken egg. The main reason that prompted the authors to carry out this study was evidence suggesting the utility of NPT in differential diagnosis of food allergy.

## Case description

A 21-year-old woman presented to the allergology clinic because of nausea, vomiting, abdominal pain, and itching of the skin each time after she ingested a chicken egg; symptoms had been present for 1 year. She also reported that two months before her visit there was an episode when 10 min after eating a raw chicken egg (in steak tartare) she started to experience severe abdominal pain, nausea, and severe itching of the skin all over her body; and she noticed hives with itchy wheals on her abdomen, chest, and lower limbs accompanied by facial redness as well as feeling of weakness and anxiety. The patient provoked vomiting and took an antihistamine drug in the form of a tablet, the name of which she could not remember. Within an hour, the symptoms resolved completely. Since then, the patient has eliminated chicken eggs from her diet. Her history revealed that she is a second-year psychology student, has an active lifestyle, practices yoga, does not use stimulants, and is interested in healthy nutrition. There is no history of allergic disease in the patient’s family. The patient had no history of chronic diseases and did not take any medications chronically. She had a history of diarrhea and spitting in infancy; at the age of 3 months she was diagnosed with cow's milk protein allergy, and thus was given milk replacer with a high degree of protein hydrolysis up to the age of 12 months. Currently, the patient fully tolerates dairy products. Due to her mother’s concerns, the diet was extended to other foods quite late, after the age of 12 months. In the 12th month of life, chicken egg yolk was introduced, which was well-tolerated; however, introduction of chicken egg white caused redness of the skin around the mouth accompanied by diarrhea. Until the age of 5, the patient did not consume any egg protein, and in the following years its consumption was heavily limited. Only at the age of 20, due to the patient’s belief about the high nutritional and health value of chicken eggs, she introduced this product into her diet in a large amount. It should be noted that initially the patient did not link her complaints with the consumption of eggs, and because of recurrent diarrhea and abdominal pain, she underwent full gastroenterological workup (including gastroscopy and colonoscopy), which ruled out gastrointestinal disorders.

## Physical examination and differential diagnosis

In our clinic, we conducted a full physical examination, with particular emphasis on the laryngological examination, and noted no substantial abnormalities. We performed skin prick tests (Allergopharma). In the food panel (cow’s milk, chicken egg, peanuts, hazelnuts, wheat flour, rye flour, celery, cod, beef, chicken meat, positive control, negative control), we found positive results only for chicken egg (wheal 4 mm, erythema 8 mm) and positive control (histamine)—wheal 7 mm, erythema 10 mm; and for the negative control—wheal 0 mm, erythema 0 mm. The skin prick tests for inhalant allergens were negative. We also obtained the sIgE panel (Polycheck) with the following results: chicken egg (protein, f01)—3.1 kU/l—class 2, and sIgE for chicken egg (yolk, f75)—class 0. The sIgE results for other allergens were negative. We also performed molecular tests, and used the ALEX multiplex test. We found high levels of IgE for nGal d 1 (ovomucoid)—7.09 kUA/L and nGal d 2 (ovoalbumin)—6.25 kUA/L; and negative IgE for nGal d 3 (ovotransferrin)—0.1 kUA/L, nGal d 4 (lysozyme)—0.1 kUA/L and nGal d 5 (serum albumin)—0.1 kUA/L; otherwise, low IgE level for nBos d 8 (casein)—0.4 kUA/L and nBos d 4 (alpha-lactalbumin)—0.35 kUA/L. The results for other extracts and molecules were negative. The total serum cIgE was 78 kU/L. There was no functional bronchial dysfunction (FEV1%VCmax 93%, SR-0.85, p20; FEV1% FVCex 98%, SP-0.30, p 38; FEV1 85%, SR-1.085; PEF 85% SR-1.12, FVCex 93%, SR-0.56; FVCin 98%, SR-0.14). Based on those studies, we initially diagnosed allergy to chicken egg protein, but to confirm this diagnosis, we qualified the patient for the provocation test. It is also important that the patient needed clear evidence of her allergy, because the need to completely withdraw chicken eggs from her diet was difficult for her to accept.

## Provocation test: nasal and food test with chicken egg allergen

The National Science Center Miniature-5 (2021/05/X/NZ5/01/099) financially supported the study, approved by the Bioethics Committee at the Medical University of Warsaw (KB/63/2022). In the provocation tests with an allergen, we conducted NPT and DBPCFC in accordance with the European Academy of Allergy and Clinical Immunology position paper on the standardization of nasal allergen challenges as well as Standardizing double-blind, placebo-controlled oral food challenges: American Academy of Allergy, asthma & Immunology—European Academy of Allergy and Clinical Immunology PRACTALL Consensus Report [8, 9] at the hospital by qualified medical personnel.

**Fig. 1:**
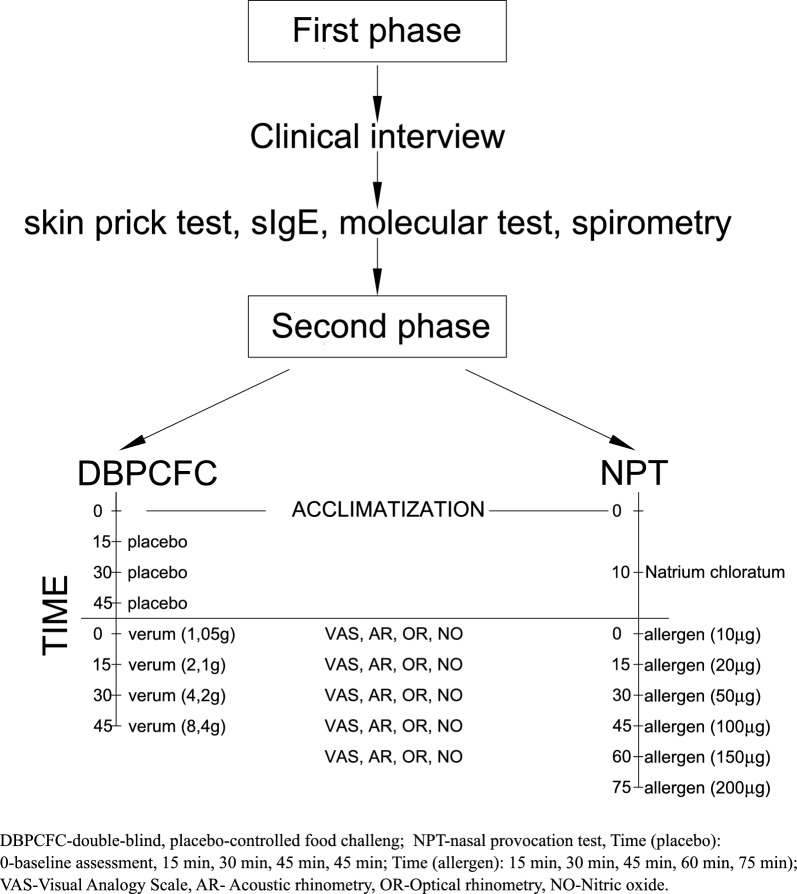
Flow chart of provocative trials

To assess the nasal mucosal response during NPT and DBPCFC, we used AR (Rhinometrics, Denmark), where we assessed the minimal cross area (MCA-1, MCA-2) on the rhinomenometric curve at the nasal vestibule and nasal concha (minimal cross area (MCA-2) on the rhinomenometric curve). OR (GmbH Rhios) directly measures changes in nasal airway patency (optical density OD), together with the nitric oxide level in the exhaled air (ProVita) and the visual analogue scale (VAS; nasal symptoms such as itchy nose, watery discharge, and nasal obstruction assessed from 0—no symptoms, to 10—most severe symptoms). In NPT and DBPCFC, we observed the early allergic reaction stage, evaluated at intervals of 15 min after increasing dose of allergen in accordance with the adopted study regimen [8, 9] (Fig. 1). In the provocation tests, we used the same allergen extract as the starting base in the form of dry chicken egg protein (energy content in 100 g–total fat 39.0 g). The extract of dry chicken egg protein powder contained all diagnostically pertinent allergen components in the following proportions: ovomucoid (Gal d 1)—11%, ovoalbumin (Gal d 2)—54%, ovotransferrin (Gal d 3)—12% and lysozyme (Gal d 4)—3%. In the titrated NPT, we prepared the doses in laboratory conditions: we weighted 2.0 mg of egg protein powder and dissolved it in 40 mL of aqueous NaCl. Such a solution contained 2000 µg of protein. The final dose of the allergen used in the titrated provocation test was based on a typical dilution scheme, in which we measured the necessary amount of the solution from the baseline solution; i.e., we extracted 0.2 mL for a dose of 10 µg; 20 µg—0.4 mL; 50 µg—1.0 mL; 100 µg—2.0 mL; 150 µg—3.0 mL; 200 µg—4.0 mL of the baseline solution. We administered the allergen in a dissolved dry substance with an atomizer to both nostrils, administering a dose of 0.1 mL each time.

In DBPCFC, the nutritionist prepared the product (allergen) in accordance with a standardized recipe for gingerbread muffin {placebo: 42 g fine white sugar, 8.5 g margarine (without dairy products), 59 g wheat flour, 8.5 mL soy cream (in the original Alpro Soja recipe), 34 mL rice milk, 0.17 g salt, 3.4 g of mixed gingerbread spice, 8.4 g of dried coconut, 10.1 g sugar syrup and verum (active sample): 8.4 g of dried whole egg powder and 50.5 g of fine white sugar, 8.5 g of margarine (without dairy products), 42 g wheat flour, 42 mL rice milk, 0.08 g salt, 8.5 g of mixed gingerbread spice, 6.7 g dried coconut, 6.7 g sugar syrup [10, 11]. We gave increasing doses of the allergen during the provocation tests at 20-min intervals (Fig. 1), and depending on the stage of the test we made measurements by objective techniques as well as the subjective VAS scale. We deemed NPT to be positive when there was a decrease of the nasal cross-section for CA-2 (treated interchangeably—MCA2) of  ≥ 40% in RA and ≥ 50 mm on the VAS scale [9]. During DBPCFC, we routinely evaluated the patient’s clinical condition and measured the pulse as well as blood pressure, auscultated the chest, and assessed the skin; we treated any abnormalities (depending on their severity) as a positive result of the provocation test. The qualification for NPT and DBPCFC involves evaluation of indications and contraindications in accordance with the recommended standards [8, 9]. We carried out both tests at an interval of 2 weeks. On the day of the study, the patient presented in good overall condition, with the following vital parameters: HR 72 bpm, BP 125/80 mmHg, Sat O_2_ 97%.

**Fig. 2:**
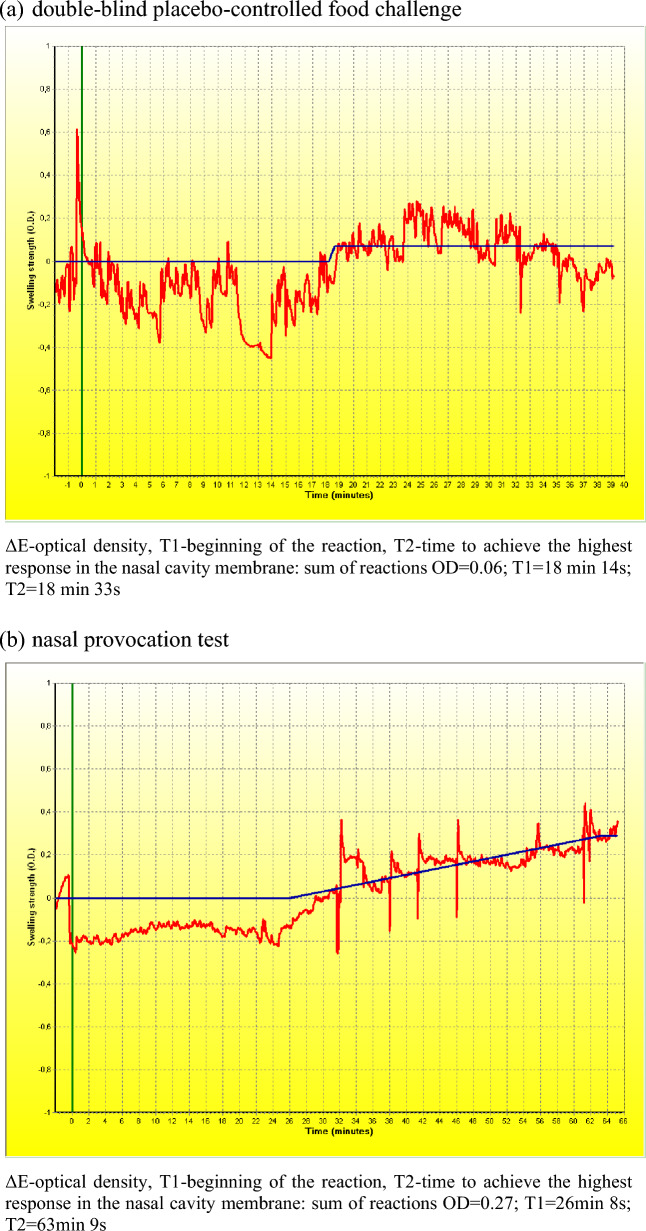
Optical rhinometry in provocation test

**Fig. 3:**
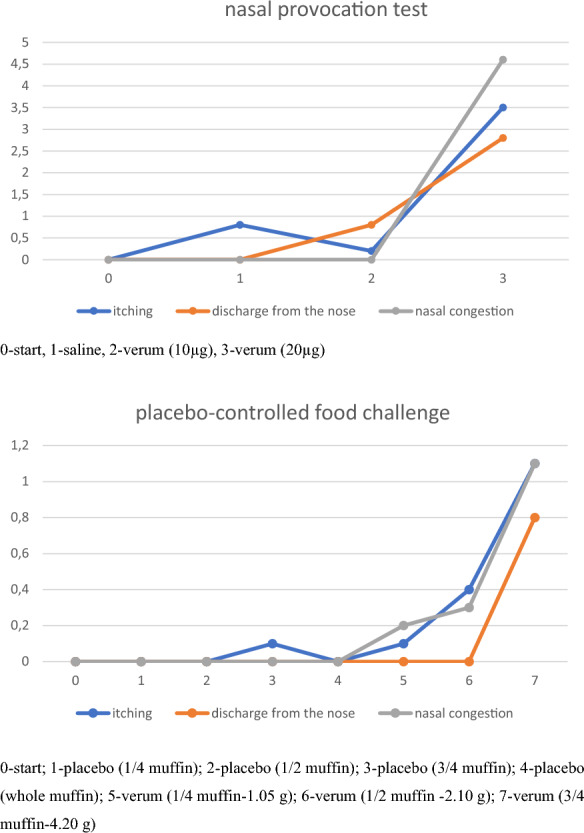
Visual analogue scale during the provocation tests

In the provocation tests, both NPT and DBPCFC, the variability of the nasal cross-sections varied with the dose of the allergen. During DBPCFC, the rate of response of the nasal mucosa was below the moderate level with a positive oral provocation test. Although the first two doses of the muffins did not cause any discomfort, after the third dose of 4.20 g the patient complained about abdominal pain and nausea. We also observed a slight increase in heart rate and blood pressure (89 bpm; 112/78 mmHg, Sat O_2_ 97%) and respiratory symptoms, including dyspnoea. We discontinued the test and deemed it positive. On the other hand, during NPT at a dose of 10 µg, there was a moderate decrease in nasal patency measured by AR, OR, and nitric oxide. The successive dose of the allergen (20 µg) in accordance with the adopted regimen resulted in a positive response of the nasal mucosa in a substantial manner; both in objective tests [AR and OR (Table 1, Fig. 2)] and the subjective VAS scale (Fig. 3). Especially in OR, we observed variability of cross-sections in the early reaction phase NPT, respectively: in 5 min ΔE =  − 0.22, T = 1 s (0:01), T2 = 2 s (0:02); 10 min ΔE =  − 0.20, T = 2 s (0:02), T2 = 5 s (0:05); 15 min ΔE =  − 0.18, T = 4 s (0:04), T2 = 8 s (0:08); 20 min ΔE =  − 0.16, T = 1 s (0:01), T2 = 6 s (0:06); 25 min ΔE =  − 0.16, T = 6 s (0:06), T2 = 13 s (0:13); 30 min ΔE =  − 0.15, T = 10 s (0:10), T2 = 20 s (0:20); 35 min ΔE =  − 0.11, T = 6 s (0:06), T2 = 18 s (0:18); 40 min ΔE =  − 0.09, T = 10 s (0:10), T2 = 23 s (0:23); 45 min ΔE = 0.13, T = 1910s (31:50), T2 = 1939s (32:19); 50 min ΔE = 0.15, T = 1912s (31:52), T2 = 1944s (32:24); 55 min ΔE = 0.15, T = 1910s (31:50), T2 = 1945s (32:25) and 60 min ΔE = 0.17, T = 1908s (31:48), T2 = 1947s (32:27).

**Table 1:** Mucosal reactivity in the provocation test

NPT									
Test stage/parameters	RA								NO
	MCA1 (0 to 2-cm distance)		MCA2 (2 to 6-cm distance)		R%_MCA1 (0- to 2-cm distance)		R%_MCA2 (2- to 6-cm distance)		Nose
	Right	Left	Right	Left	Right	Left	Right	Left	
0	0.255	0.342	1.474	1.571	2	4	1	1	931 ppb
NaCl (control solution)	0.250	0.355	1.462	1.595					920 ppb
10 µg	0.211	0.336	1.178	1.364	16	5	20	14	901 ppb
20 µg	0.209	0.325	1.065	1.007	16	8	27	36	867 ppb
DBPCFC									
0	0.267	0.384	1.511	1.498	3	6	3	1	986 ppb
Placebo (1/4 muffin)	0.259	0.362	1.464	1.511					986 ppb
Placebo (1/2 muffin)	0.250	0.373	1.450	1.516	6	3	4	1	984 ppb
Placebo (3/4 muffin)	0.256	0.366	1.451	1.480	4	5	4	1	979 ppb
Placebo (whole muffin)	0.251	0.354	1.449	1.466	6	8	4	2	981 ppb
*Verum* (1/4 muffin 1.05 g)	0.245	0.336	1.385	1.422	8	12	9	5	975 ppb
*Verum* (1/2 muffin 2.10 g)	0.239	0.333	1.378	1.309	10	13	9	13	974 ppb
*Verum* (3/4 muffin 4.20 g)	0.231	0.319	1.345	1.296	13	17	11	13	971 ppb

0-baseline assessment, MCA1-minimum cross-section area measured at the vestibule of the nasal cavity, expressed in cm^2^; MCA2-minimum cross-sectional area measured at the head of the nasal concha; re%_MCA1-reactivity expressed as a percentage of MCA1, expressed in cm^2^; re%_MCA2-reactivity expressed as a percentage of MCA2; NO-nitric oxide

Due to the positive reaction to the allergen, we discontinued the test. We observed no other symptoms during NPT such as dyspnoea, which is reflected by the stable level of nitric oxide in the exhaled air. The patient remained under observation. We discharged her home 48 h after administration of the allergen, and advised her to limit her intake of chicken eggs.

## Discussion

In our paper, we present the case of a patient with food allergy to chicken egg protein, in whom we used NPT for the diagnosis, and we observed a response of the nasal mucosa during DBPCFC. Our study is a response to the consensus by the European Academy of Allergy and Clinical Immunology position paper on the standardization of nasal allergen challenges [8], in which emphasis is on on the possible broader application of the nasal test, such as NPT being a component of differential diagnosis of food allergy. Our selection of objective techniques for assessment of nasal patency used in the provocation tests was not accidental and included RA as well as nitrogen oxide analysis in the exhaled air. RA is an excellent tool based on the recording of transverse section / volume with acoustic sound and enables monitoring the response of the nasal mucosa during NPT. On the other hand, nitric oxide is a sensitive marker of an inflammatory response, and tends to decrease with a strongly positive NPT (although in the early stage of the allergic reaction it increases) due to blockage of the nasal passages, which blocks access to the target site of nitric oxide production; i.e., the paranasal sinuses.

Allergy to chicken egg white is one of the most common forms of food allergy in allergology practice. The incidence of chicken egg allergy ranges from 0.5 to 2% in infants and young children [12]. A multicenter cohort study Europrevall conducted between 2005 and 2009 in nine European countries indicates that the incidence of chicken egg allergies in children up to the age of 2 is 1% [13]. Food allergy to chicken eggs in the adult population is much less common and affects 0.1%. It is believed that in adults, persistent allergy predominates; and a new-onset allergy to egg protein is rare [14]. Food allergens are mainly glycoproteins with a molecular weight of 15–50 kD. The allergenicity of molecules depends on the number of epitopes to which specific antibodies can attach. The epitope structure determines the maintenance or loss of allergenic properties [1, 15]. Allergy to egg allergens is usually related to the allergy to chicken egg protein, especially to ovomucoid (Gal d 1) and ovalbumin (Gal d 2). Ovomucoid retains allergen activity even after cooking for an hour, and only higher temperatures (‘baked egg’) reduce the allergenicity of ovomucoid [15, 16]. Some data indicate that baking eggs together with wheat contributes to a reduction of the allergenicity of ovomucoid [10]. Owoalbumina and transferrin (Gal d 3) are partially resistant to digestion and thermolabile. Lysozyme (Gal d 4) is a preservative of many food products. Allergy to the Gal d 5 (alpha-livetin, serum albumin) contained in egg yolk may cause a cross-reaction with the chicken meat (so-called *bird-egg syndrome)* [15].

Allergy to chicken eggs is particularly difficult for patients and their families, due to the need to avoid many foods and fear of accidental ingestion; it is also an unfavorable prognostic factor of future allergy to inhalant allergens and development of asthma as well as allergic rhinitis [16]. For this reason, performing an accurate diagnostic process is important, and the safety of the diagnostic procedures should be considered as well. Currently, despite intensive development of molecular methods in the diagnosis of food allergy, the gold standard and the most reliable method remains the oral provocation test. It is of particular importance in confirming allergy to chicken egg protein and consequently for elimination from the diet as well as exclusion of other diseases requiring a treatment other than simply diet. However, when deciding on the provocation test, the limitations of this method should be considered; such as possible anaphylaxis or other serious reactions, possible false negative and false positive results, and the costs associated with performing a provocation test at the hospital [10, 11]. Based on those observations, there is an ongoing search for safer, more accessible, and outpatient diagnostic tests. In 2013, Kvenshagen and Jacobsen emphasized in their work the necessity to search for new diagnostic methods in food allergy due to its increase in morbidity; considering the potential risk, high costs, and time consumption of the oral provocation test [7]. Based on a literature review, the authors described the possibility of utilizing mucosal allergen challenge in the diagnosis of food allergy. Due to the accessibility of the mucous membrane and the possibility of using small allergen doses, they evaluated those methods as promising. In 1985, Amlot et al. presented a study with the use of nasal, lip, and gastric provocation test on 39 patients with diagnosed milk and chicken egg allergy, based on history and positive skin prick tests. They assessed the results of the nasal provocation test based on PNIF measurements and the number of sneezes. They performed no oral food challenge. Based on the results, the most sensitive test was the nasal provocation test. However, there are few published studies presenting the use of nasal provocation test in the diagnosis of food allergy [17]. In 1993 Seppey et al. [18], and subsequently in 2007 and 2012 Clark et al. [19, 20], researchers presented studies about nasal provocation tests using chicken egg and peanut allergens. For evaluation of the test results, the authors used facial thermography and considered the test to be fast, safe, and objective. Gelis et al. presented an interesting study assessing the usefulness of nasal provocation tests in the diagnosis of shellfish allergy as well as differentiation of patients with allergy and non-allergic hypersensitivity as an alternative to oral provocation tests. The study included 45 people with confirmed allergy to shrimp in the prick skin test, oral provocation tests, previous episode of anaphylaxis, or intolerance to shrimp based on history. The control group consisted of 10 healthy individuals. In the nasal test, they used lyophilizate of cooked shrimps and evaluated the results using acoustic rhinometry as well as a visual analogue scale. The results confirmed the usefulness of NPT in the diagnosis of food allergy to shrimp [21]. The work of Gelis et al. was an inspiration for our team, and in our study in 2020 we confirmed the reactivity of the nasal mucosa during the oral provocation test with cow's milk protein allergens [22]. In our current study in the diagnosis of food allergy, in addition to the recognized diagnostic method (i.e., DBPCFC), we used a new diagnostic method (NPT) with chicken egg protein, and confirmed its effectiveness and safety. The results of our study are promising for further use of NPT in the diagnosis of food allergy.

## Conclusions

The nasal provocation test seems to be a useful and safe tool in the diagnosis of food allergy, with a potential for obtaining objective final results.

## Data Availability

Data sharing is not applicable to this article as no datasets were generated or analyzed during the current study.
